# Therapeutic potential of greenly synthesized selenium nanoparticles against experimental cyclosporiasis

**DOI:** 10.1038/s41598-025-15238-8

**Published:** 2025-08-17

**Authors:** Nehal N. Hezema, Marwa M. Eltarahony, Heba Afifi, Sara A. Abdel Salam

**Affiliations:** 1 Department of Medical Parasitology, Faculty of Medicine, Alexandria University, Alexandria, Egypt; 2 Department of Environmental Biotechnology, Genetic Engineering and Biotechnology Research Institute, City of Scientific Research and Technological Applications, New Borg El‑Arab City, Alexandria Egypt; 3 Department of Pathology, Faculty of Medicine, Alexandria University, Alexandria, Egypt

**Keywords:** *Cyclospora cayetanensis*, Selenium nanoparticles, Green synthesis, Scanning electron microscopy, Anti-inflammatory, Antioxidant, Nanobiotechnology, Nanoparticles, Parasitology, Nanoscience and technology, Gastrointestinal diseases, Infectious diseases

## Abstract

*Cyclospora cayetanensis (C. cayetanensis)*, an opportunistic intracellular coccidian, is responsible for chronic debilitating diarrheal outbreaks possessing life-threatening penalties, especially in immunocompromised patients. The solemn therapeutic agents against cyclosporiasis are limited by their grave effects and high recurrence rate. The current study aimed to utilize greenly synthesized selenium nanoparticles (SeNPs) and evaluate their therapeutic efficacy on cyclosporiasis in immunosuppressed murine models. They were biosynthesized proficiently using *Alcaligenes faecalis* and characterized by assorted analytical techniques. After molecular confirmation of the parasite strain, immunosuppressed mice were infected with 10,000 *C. cayetanensis* sporulated oocysts. The anti-*Cyclospora* activity of seven-day oral treatment of 10 mg/kg of SeNPs was assessed through parasitological, ultrastructural, histopathological, and biochemical studies. The in vivo safety of SeNPs was assessed clinically, biochemically, and histopathologically. Parasitologically, SeNPs recorded the highest statistically significant decrease in the fecal oocyst burden (97.96%R) on the 30th day post-infection (PI). Scanning electron microscopic examination revealed remarkably deformed SeNPs-treated oocysts. SeNPs-treated mice exhibited impressive amelioration in intestinal architecture and inflammation, protracted to the 30th day PI. Colorimetric analysis revealed that SeNPs have recorded the highest serum reduced glutathione (GSH) level (300% increase) that swiftly repressed malondialdehyde (MDA) by 63.46%R. The present work has shed the first light on biogenic SeNPs as a safe, promising, proficient antioxidant nanotherapeutic for the treatment of experimental cyclosporiasis.

## Introduction

Human cyclosporiasis is an emerging, ubiquitous water- and food-borne disease concerning the human health and ecosystem^1^. *Cyclospora cayetanensis* (*C. cayetanensis*), *C. ashfordi*, and *C. henanensis* are the three known causative agents of human cyclosporiasis^2^. The opportunistic coccidian, *C. cayetanensis*, is the most common human-infecting species^1^. The existence of animals as natural reservoirs of *C. cayetanensis* has not yet been confirmed. However, successful attempts to establish *C. cayetanensis* infection in laboratory guinea pigs, Swiss albino mice, Asian freshwater clams, and oysters have been documented^3^. Human cases of *C. cayetanensis* infections have been recorded in 54 tropical and subtropical countries, and 13 of them have reported seasonal outbreaks^1,4^. Children, immunosuppressed patients, and expatriates in endemic countries are more prone to *C. cayetanensis* infection than the indigenous population, while any age group could be infected in developed countries^5^. Although the disease in most of the infected patients is self-limiting, immunosuppressed patients may present with severe protracted explosive watery diarrhoea, and even extra-intestinal organ colonization, causing cholecystitis^5^.

To date, no effective vaccine exists for human cyclosporiasis. Co-trimoxazole (CMX), a combination of trimethoprim (TMP) and sulfamethoxazole (SMX), is the most effective prescribed treatment^6^. However, the serious apparent side-effect profile of the afore-mentioned treatment encompasses mainly myelosuppression, hepatotoxicity, nephrotoxicity, and hypersensitivity to sulfa have been reported^6,7^. Although Ciprofloxacin and nitazoxanide are less effective than CMX, they proved to be suitable alternatives for infected individuals intolerant to CMX. Besides the long-term treatment duration, the reported drug resistance had, ultimately, led to a high rate of recurrence^6^. Development of an efficient nontoxic biocompatible therapeutic agent against cyclosporiasis to overcome the emerging shortcomings of the current established treatment is of utmost importance.

Nanotechnology-based therapies constitute an efficient, substantial solution to address the emerging pressing problem of drug resistance due to the outstanding physical and chemical properties of nanoparticles (NPs) over conventional treatments^8^. With the advent of metal-based NPs, they have been accredited in parasitological research, owing to their distinct physicochemical merits. Despite the tremendous antiparasitic activities witnessed by mechanochemically synthesized metal NPs against a myriad of diseases, the generation of harmful toxic reagents affecting both human health and the environment was reported^9^.

Green nanotechnology, the new generation of nanomedicine, has been propelled to the forefront in nanotherapeutics. It is a set of eco-friendly, sustainable, and greener alternatives offering safe, highly biocompatible, and inexpensive methods that eliminate the utilization and generation of hazardous chemicals^10^. Several biological entities, plants, actinomycetes, fungi, algae, yeast, and bacteria, have been exploited in the green fabrication of NPs^11,12^. Bio-inspired green metal NPs such as gold, silver, platinum, copper, titanium, iron, zinc, and selenium (Se) have been successfully employed for the treatment of diverse parasitic diseases^13,14^.

The essential micronutrient, Se, plays a pivotal role in versatile physiological and metabolic regulatory signalling processes in both prokaryotes and eukaryotes^15^. The pro/anti-oxidant activity, bioavailability, and toxicity of Se depend on its chemical form. Inorganic and organic Se compounds are associated with low redox activity. Besides its narrow safety-to-toxicity margin, inorganic selenium salts have high cytotoxicity and are difficult to absorb^16^. On the other hand, SeNPs exhibit diverse pharmacokinetic advantages such as high metabolic stability, membrane permeability, biological and antioxidant activity, as well as low toxicity, compared to its organic and inorganic counterparts. Additionally, they provide a depot formulation of Se through the gradual release of dissolved Se from the particles’ surface^17^.

Biogenic SeNPs possess more biocompatibility and better stability than chemical synthesis. Besides being free from toxic/hazardous components, the use of natural organisms provides a reliable, simple, low-cost, and eco-friendly method. Compared with organic and inorganic selenocompounds such as sodium selenite, SeNPs possessed significantly reduced risk of Se toxicity, increased bioavailability, and stronger activity^16–18^. As reported by Sonkusre 2020, upon SeNPs treatment, an increase in the total liver Se indicated the absorption of particles and minor liver toxicity. H&E-stained sections of liver, kidney, and spleen of mice challenged with 50 mg Se/kg SeNPs for 10 days showed no toxicity or morphological changes in liver and kidney tissue^19^.

The use of Se as a potential nanotherapeutic agent has revolutionized the biomedical field^20^. A large body of evidence supports higher efficiency, biocompatibility, and negligible side effects of greenly synthesized SeNPs than their chemical counterparts^10^. The biosynthesis of NPs using aerobic Se-reducing bacteria is simple, cheap, fast, stable, safe, and eco-friendly^21^. To name a few, bio-synthesized SeNPs were used to combat diabetes^22^cancer^23^Parkinson’s disease^24^rheumatoid arthritis^25^and cognitive dysfunction^26^. Apart from their high therapeutic potentials in non-infectious diseases, greenly synthesized SeNPs possess antibacterial^27^ and antifungal activities^28^. Concerning parasitic diseases, several in vivo studies have investigated the enhanced therapeutic activity of biogenic SeNPs against cystic echinococcosis^29^acute and chronic toxoplasmosis^30,31^cutaneous leishmaniasis^32^and eimeriosis^33^.

Based on the great potency of biogenic SeNPs against relevant parasitic diseases, they have been identified as a treasure trove in preclinical studies. The green fabrication of SeNPs using *Alcaligenes faecalis* could change the trajectory of cyclosporiasis therapy, especially in immunocompromised and is worth studying. Thereby, the objective of the current study was to evaluate the therapeutic potential of biosynthesized SeNPs against cyclosporiasis in immunosuppressed murine models via parasitological, ultrastructural, histopathological, and biochemical studies.

## Materials and methods

### Drugs

Cyclophosphamide (Endoxan, Asta Medica AG, Germany) was used as an immunosuppressive agent in weekly intraperitoneal doses of 70 mg/kg each^34^. Cotrimoxazole (CMX) (Sutrim suspension, Memphis Co. for Pharm. & Chem. Ind., Egypt) was given to mice orally at a dose of 5 mg/kg TMP in combination with 25 mg/kg SMX once daily^35^.

## Biosynthesis and characterization of SeNPs

The bacterial strain *Alcaligenes faecalis*, a marine isolate, was screened from the Mediterranean Sea water, El-Max district, Alexandria, Egypt. This strain, designed as 46 N, displayed promising biochemical and physiological performance, especially in denitrification and NPs synthesis processes under oxic/anoxic conditions. Its 16 S ribosomal deoxyribonucleic acid (rDNA) gene sequence was submitted to the GenBank under the accession number of KY995586. For biosynthesis process, the bacterial lawn (0.5 McFarland ≈ 10^8^ CFU/ml) was cultured in 500 mL flasks containing 120 mL of nutrient broth (NB) composed of the following (g/L): peptone 10, beef extract 1, yeast extract 5, NaCL 5, pH 7 and incubated for 24 h at 30 °C under orbital shaking incubation (150 rpm). After complete incubation, the bacterial biomass was harvested, under aseptic conditions, by centrifugation (12,000×*g*) for 10 min. The obtained aqueous filtrate of *Alcaligenes faecalis* was sterilized using a syringe filter (0.22 μm pore size) and homogenously mixed with sterile 1.5 mM of Na_2_SeO_3_. Further, as a control, flasks were prepared with NB media inoculated with 2 mM of Na_2_SeO_3_ in parallel to NB media inoculated with bacteria and devoid of Na_2_SeO_3_ incubated under exact conditions. The biotransformation of Na_2_SeO_3_ to SeNPs was monitored visually and regularly during the incubation period. The obtained SeNPs were collected by centrifugation at 12,000×g for 20 min, washed twice by 70% ethyl alcohol and double-distilled H_2_O to remove any remaining. Afterwards, SeNPs were sterilized by filtration (syringe filter 0.22 μm), followed by endotoxin evaluation, yielding a measured limulus amebocyte lysate (ALA) value of < 0.06 EU/mL. Eventually, the biosynthesized SeNPs were dried at 80 °C for 5 h for characterization^30^.

The optical properties with correlated surface plasmon resonance (SPR), while identity, crystalline phase, and morphological properties of biosynthesized SeNPs were scrutinized by a Labomed model UV-Vis double-beam Spectrophotometer, X-ray diffractometer (XRD) (Shimadzu 7000, USA), and transmission electron microscopy (TEM) (JEOL JEM-1230), respectively. However, the elemental compositions and profile of functional groups were determined using an energy dispersive X-ray spectroscopy (EDX) (JEOL JSM-6360LA) and Fourier-transform infrared spectroscopy (FTIR) (Shimadzu FT-IR-8400 S, Japan), respectively. Moreover, the Dynamic light scattering technique (DLS) was employed to determine the particle size distribution (PSD) profile, polydispersity index (PDI) and zeta potential through nano-zetasizer (Malvern Instruments, Worcestershire, UK) with a scattering angle of 90° at a temperature of 25 °C^30^.

### Isolation and maintenance of *C. cayetanensis* oocysts

The oocysts were isolated and concentrated from sieved stool samples of heavily infected HIV patients by 12-min centrifugation at 2000 rpm^36^. The concentrated samples were examined for the presence of *C. cayetanensis* oocysts by staining with both modified Ziehl-Neelsen (MZN) and safranin stains. Then, the isolated oocysts were preserved in 2.5% potassium dichromate solution at 4 °C^37^.

### Molecular characterization of *C. cayetanensis*

The qualitative real-time polymerase chain reaction (qRT-PCR) with SYBR Green dye and DNA melting curve analysis was performed to confirm the diagnosis of the isolated *C. cayetanensis* oocysts^38^. DNA was extracted using QIAamp DNA Mini Kit (QIAGEN, GERMANY) according to the manufacturer’s protocol. PCR amplification was performed using *C. cayetanensis*-specific forward (5’-TAGTAACCGAACGGATCGCATT-3’) and reverse (5’-AATGCCACGGTAGGCCAATA-3’) primers^39^.

## Experimental design

### Animals

Swiss albino male mice, four to six weeks weighing about 23 ± 2 g, were obtained from the animal house of the Medical Parasitology Department, Faculty of Medicine, Alexandria University, Egypt and kept in vivarium according to standard breeding conditions (25 ± 2 °C and 12 h light/dark photoperiod) with free access to food and water.

### Sporulation of *C. cayetanensis* oocysts

Oocyst sporulation was achieved by putting the positive potassium dichromate preserved samples in covered Petri dishes at room temperature (about 25 °C) for 8–14 days. Daily microscopic examination of oocysts was performed to ensure the occurrence of sporulation. Subsequently, sporulated oocysts were used for animal infection^35^.

## Animal infection

After being centrifuged at 1500×g for ten minutes and washed with sterile phosphate-buffered saline (PBS) three times to remove the potassium dichromate, the sporulated *Cyclospora* oocysts were counted using a haemocytometer and administered orally at a dose of 10^4^ oocysts/0.1 ml of PBS per mouse for induction of the infection^35^.

### Animal grouping

A total of 42 mice were divided into four groups as follows: Group I, six non-infected non-treated mice; Group II, 12 infected non-treated mice; Group III, 12 infected CMX-treated mice; and Group IV, 12 infected SeNPs-treated mice.

Mice of groups II, III, and IV were infected with *C. cayetanensis* oocysts 48 h after the second dose of cyclophosphamide. Treatment was initiated on the 6th day post infection (PI) and continued for seven successive days. Based on prior reliable toxicity and parasitological efficacy studies, the biosynthesized SeNPs were administered to mice in a dose of 10 mg/kg/day. The latter dose previously proved its biochemical safety regarding liver and renal markers and induced the highest reduction in brain-cyst burden of *Toxoplasma gondii*, an intracellular coccidian^31,32^.

Six animals from each infected group were sacrificed on the 14th day PI for assessment of the efficacy of therapy. The remaining mice of the infected groups were sacrificed on the 30th day PI for assessment of recurrence. Each mouse was anesthetized with an intraperitoneal injection of 40 mg/kg of pentobarbital sodium before collecting a blood sample from the jugular vein. Thereafter, they were euthanized using cervical dislocation.

### Assessment of anti-*cyclospora* activity

#### Parasitological study

Individual stool samples were collected from each mouse in each infected group on the 6th, 10th, 14th, and 30th days PI. Four fresh stool smears (50 µl/smear) were stained with MZN and safranin stains, two smears of each stain, for each infected mouse^40^. The faecal oocyst burden in each mouse was counted in ten high-power fields (x 400) per smear. Then, the mean number of oocysts in each mouse was calculated. Then, the mean faecal oocyst count was estimated for each infected group^37^.

#### Ultrastructural study

Stool sediments containing the shedding oocysts obtained from mice in the infected groups were fixed in 2.5% buffered glutaraldehyde phosphate, dehydrated by sequential incubations in ascending concentrations of ethanol, and examined under scanning electron microscopy (SEM) (JEOL JSM, IT200, Japan)^35^.

#### Histopathological study

Ileal specimens were collected from infected mice in various studied groups on the 14th and 30th days PI. Fresh specimens were immediately fixed in 10% neutral buffered formalin, then routinely processed and embedded in paraffin. The tissue blocks were sectioned into 4–5 micrometres (µm)-thick sections and stained with hematoxylin and eosin (H&E). Histopathologic examination of the H&E-stained sections was conducted to assess architectural distortions and inflammatory infiltrate. Intestinal villus height was measured in µm by the Leica Application Suite (version 4.12.0) image analysis unit, Pathology Department, Faculty of Medicine, Alexandria University, Egypt.

#### Biochemical study

GSH and MDA levels were measured in sera of mice in all studied groups on the 14th day PI using a colorimetric kit (Biodiagnostics, Egypt) according to the manufacturer’s instructions.

#### Safety study

12 Swiss albino male mice were divided equally into two groups as follows: six non-infected non-treated mice and six non-infected mice treated orally with SeNPs in a dose of 10 mg/kg/day for seven successive days. Clinically, safety was assessed by observing the mortality rate, general health, changes in behavior, and body weight of the studied mice. Thereafter, the animals were sacrificed, and blood, ileum, liver, and kidney specimens were collected from them. Biochemically, alanine aminotransferase (ALT), aspartate aminotransferase (AST), and creatinine levels were measured in serum^31^. Histopathologically, the ileum, liver, and kidney sections were stained with H&E to assess the safety of the used dose of SeNPs.

### Statistical analysis

All data were analysed using IBM SPSS software package version 20.0. (Armonk, NY: IBM Corp). Shapiro-Wilk test was applied to test the normality of continuous data, and Levene’s test was conducted to test homoscedasticity. Quantitative data were reported as median, minimum, maximum, mean, and standard deviation. As for normally distributed quantitative variables, One way ANOVA test was used to compare the four studied groups. Then, a Post Hoc test (Tukey) was applied for pairwise comparison between groups. While Student t-test was used to compare the two main groups analyzed in Tables 4 and 5. Additionally, the Paired t-test was used to compare two different time points. Significant levels of the results obtained were expressed at the 5% level (*p* value < 0.05).

The percentage reduction (%R) and percentage of increase (% increase) were calculated consistently with the following equations:

$$\:\text{P}\text{e}\text{r}\text{c}\text{e}\text{n}\text{t}\text{a}\text{g}\text{e}\:\text{r}\text{e}\text{d}\text{u}\text{c}\text{t}\text{i}\text{o}\text{n}\:\left(\text{\%}\text{R}\right)=\frac{\text{N}-\text{n}}{\text{N}}\:\times\:100$$

$$\:\text{P}\text{e}\text{r}\text{c}\text{e}\text{n}\text{t}\text{a}\text{g}\text{e}\:\text{o}\text{f}\:\text{i}\text{n}\text{c}\text{r}\text{e}\text{a}\text{s}\text{e}\:\left(\text{\%}\:\text{i}\text{n}\text{c}\text{r}\text{e}\text{a}\text{s}\text{e}\right)\:=\frac{\text{n}-\text{N}}{\text{N}}\:\times\:100$$

N: Mean value in the infected non-treated control group.

n: Mean value in infected treated group.

## Results

### Biosynthesis and characterization of SeNPs 

#### Visual and optical properties

**Fig. 1:**
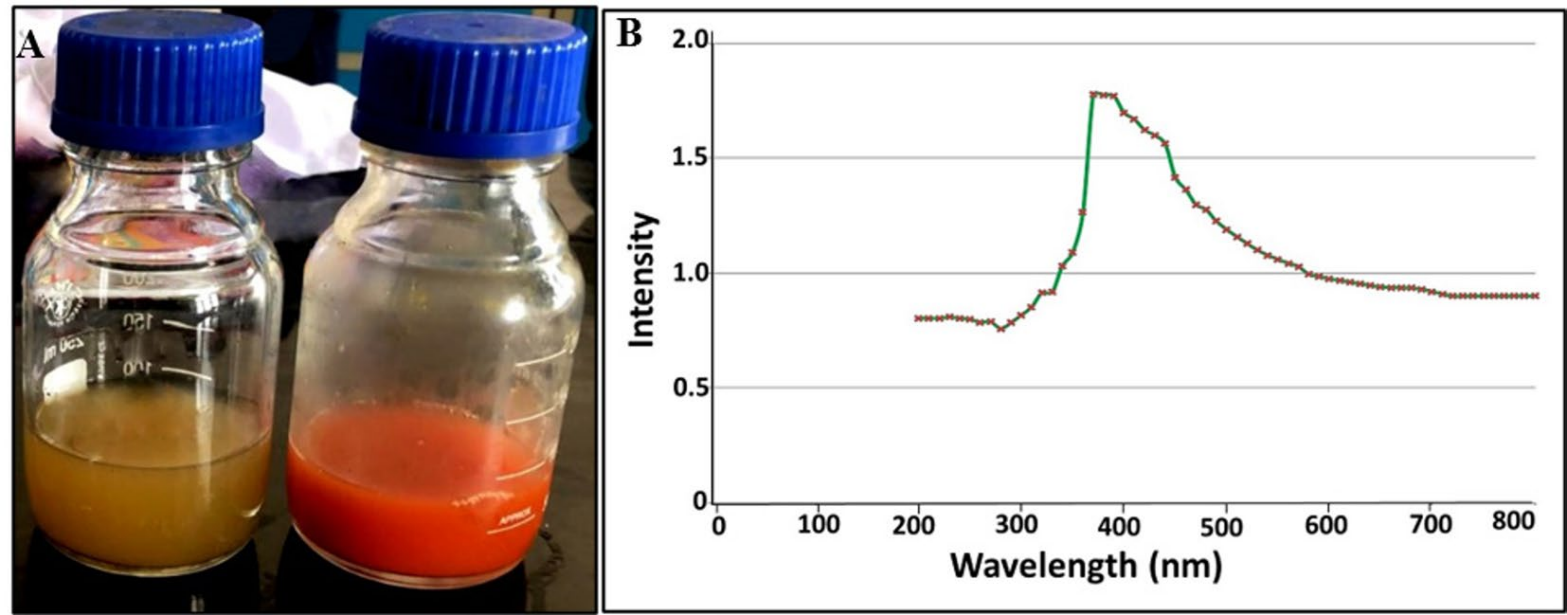
Visual monitoring (**A**) and optical properties (**B**) of bacterially synthesized SeNPs by *Alcaligenes faecalis* strain 46 N

In this study, the green preparation of SeNPs by *Alcaligenes faecalis* strain 46 N was confirmed through the development of red colour compared to the pale-yellow colour of control media (Fig. 1A), which reflected the successful bioconversion of Se precursor to its SeNPs counterpart. This observation was harmonized with the optical property of SeNPs as SPR revealed the presence of a single sharp absorption peak at 373 nm by UV-Vis spectroscopy (Fig. 1B). Our result was consistent with Pouri et al., who observed the SPR of SeNPs in a wide range from 250 to 450 nm^41^.

#### Structural properties

**Fig. 2:**
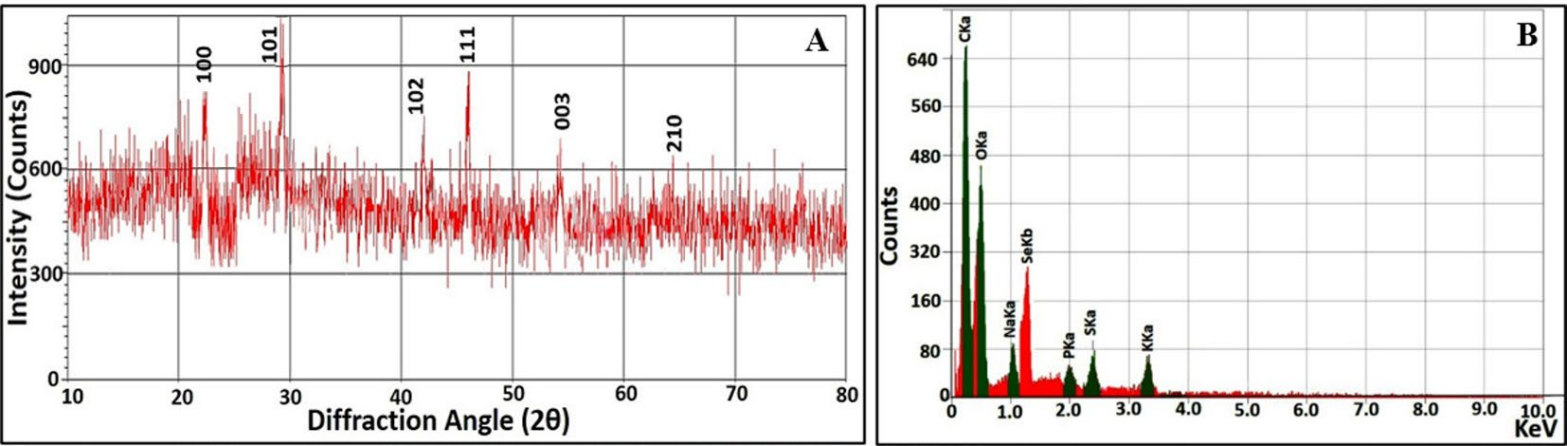
Structural and compositional properties of SeNPs synthesized by *Alcaligenes faecalis* strain 46 N: (**A**) XRD diffractogram and (**B**) EDX spectrography

As shown in Fig. 2A, diffractogram of SeNPs displayed noise background with definite peaks at 23.5°, 29.7°, 43.6°, 45.4°, 51.5° and 65.3° which matched to Miller Indices (h k l) of (100), (101), (102), (111), (201) and (210), respectively. These characteristic peaks were consistent with the standard spectrum (JCPDS, number 42-1425). Interestingly, SeNPs exhibited a crystalline and pure nature, which agreed with that obtained by Pouri et al.^41^.

In addition, the elemental composition of SeNPs via EDX analysis emphasized the involvement of Se in the sample with atomic percentages of 27.8%, indicated by the existence of a typical characteristic peak at 1.37 keV of SeLα (Fig. 2B). Besides, the characteristic signals of carbon, oxygen, phosphorus, and sulfur were also detected at 0.277, 0.525, 2.013, and 2.3 keV, respectively, highlighting the association of SeNPs with bacterial moieties concerning protein, phospholipids, nucleic acids, polysaccharides, etc. Intriguingly, the detected percentage of Se in the current study was higher than that recorded by Liang et al.^42^. who found that Se in the greenly synthesized SeNPs was 21%.

#### Morphological properties

**Fig. 3:**
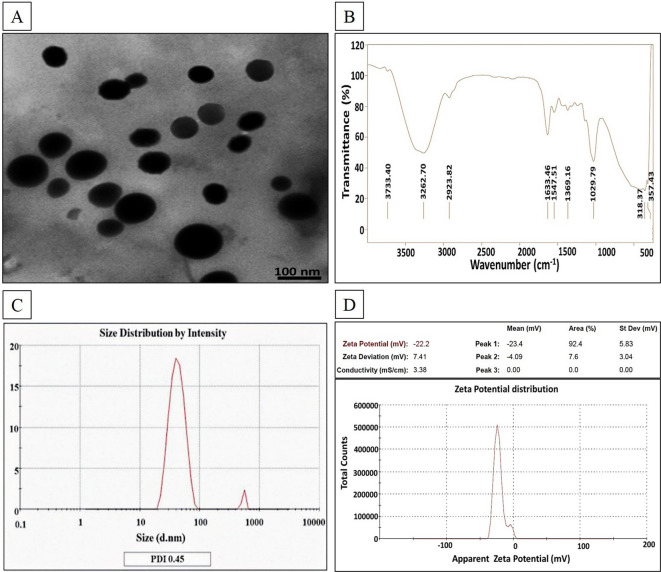
Morphological, functional, and surface charge features of SeNPs synthesized by *Alcaligenes faecalis* strain 46 N: (**A**) TEM micrograph (x10000 and accelerating voltage of 200 kV); (**B**) FTIR pattern; (**C**) PSD curve; (**D**) Zeta potential

The topographic properties, aggregation performance, and uniformity of SeNPs were depicted through TEM. Figure 3A elucidated numerous individual mono-scattering spherical-shaped SeNPs of particle size of 81 ± 15 nm, without any aggregation, implying their stabilization by bacterial entities, and agreed with that inferred from EDX analysis. Such results were consistent with those reported by several former studies^27,43^.

#### Functional properties

The functional traits of SeNPs were examined using FTIR analysis. Figure 3B portrayed the existence of some intensive bands in FTIR profile of SeNPs. Initially, there was a peak at 3733 cm^−1,^ which could be attributed to the stretching vibrations of O–H groups that are related to the absorbed water molecule. Besides, the absorption peaks at 3226 and 1547 cm^−1^ are ascribed to the amine group of proteins (N-H)^44^. Meanwhile, the existence of stretching vibrations of C-H bonds that constitute protein and lipid could be implied from the band at 2923 cm^−1 30^. However, the spectral peaks at 1633 and 1547 cm^−1^ reflected the existence of –C = C bond and amide I/II groups, respectively, as denoted by Eltarahony et al.^45,46^. Remarkably, the spectral bands at 1369 cm^−1^ could be attributed to the symmetric stretch carboxyl group (–COOH)^47^. In addition, the bands at 1029 cm^−1^ indicated the conjugation of the PO_4_^3−^ group^46^. However, Tugarova et al.^48^. stated that the bands centered in the region of 1200–1000 cm^[– 1^, implied the association of polysaccharides. Notably, Chaudhari et al.. highlighted that the inter-atomic vibrations of metal oxides were detected in the fingerprint region of the FTIR spectrum, namely below 1000 cm^−1^, which could explain the absorption band at 357 cm^−149^. Our data were in line with Arafa et al.^30^.

#### Particles surface properties

As depicted in the PSD curve, 97.0% of SeNPs were approximately 42.8 ± 12.8 nm, while about 3% were 540.2 ± 30.7 nm, which could be attributed to water and bacterial biomolecules conjugated with the SeNPs surface (Fig. 3C). Wherein, PDI index was assessed at 0.45 (Fig. 3C) and zeta-potential value was appraised by −22.2 mV (Fig. 3D).

### Molecular confirmation of *C. cayetanensis* oocysts

**Fig. 4:**
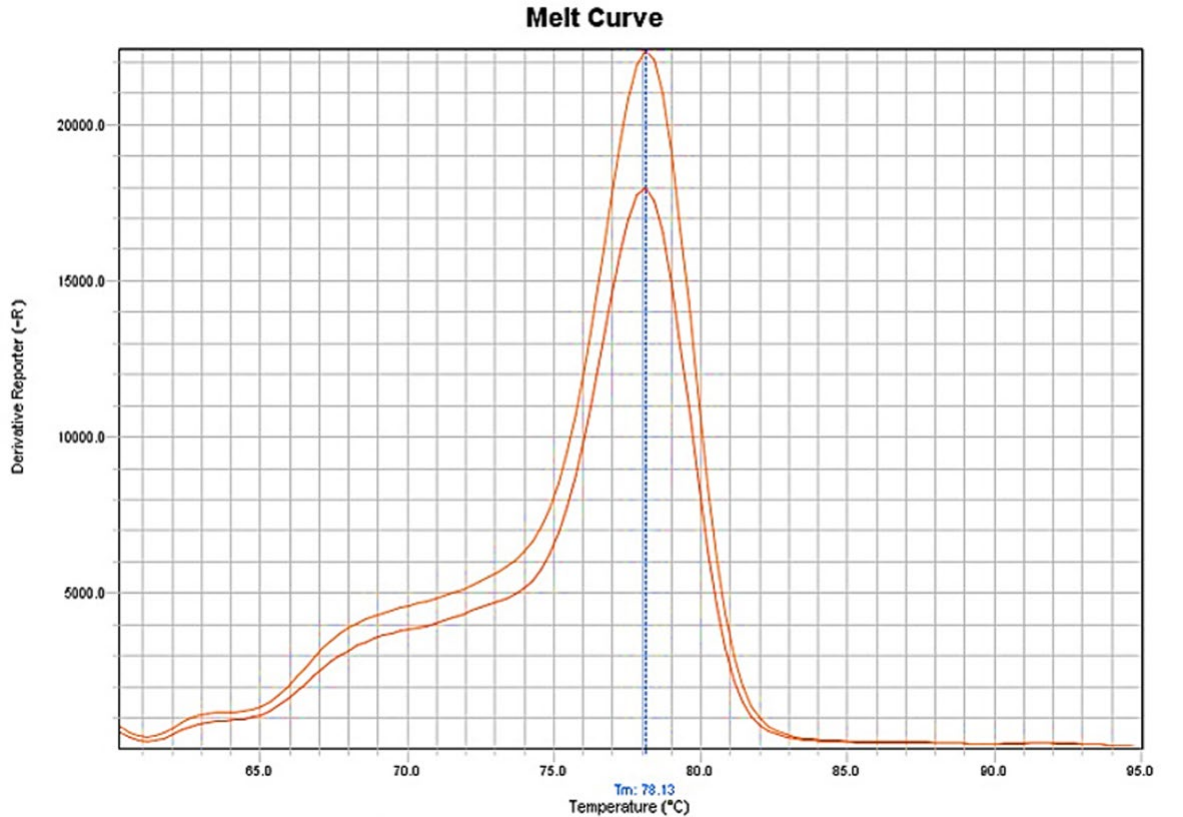
Melting curve of *C. cayetanensis* by qRT-PCR

The presence of *C. cayetanensis* oocysts was confirmed by SYBR Green qRT-PCR (Fig. 4).

#### Parasitological study

**Fig. 5:**
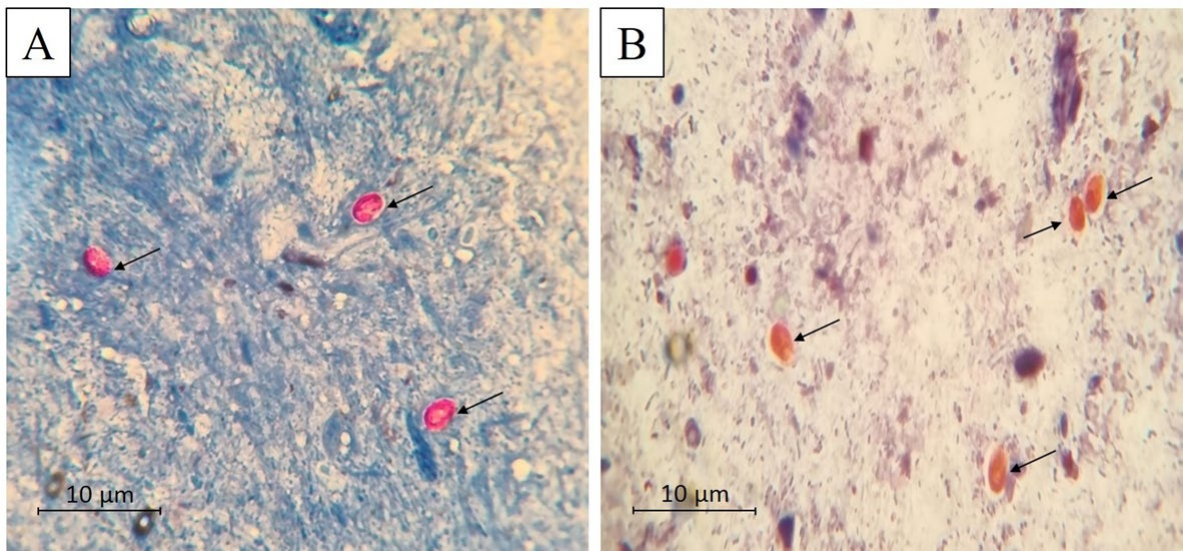
Light microscopy of *C. cayetanensis* oocysts retrieved from the stool of infected mice (*n* = 36) stained: (**A**) light pink to red with a mottled appearance by MZN stain (x1000); (**B**) orange by safranin stain (x1000)

Shedding oocysts were counted in MZN and safranin-stained faecal smears (Fig. 5). On the 6th day PI, there was a statistically nonsignificant difference in oocyst count among all studied infected groups. After treatment initiation, a statistically significant reduction in the mean parasite count was detected in both infected treated Groups (III and IV) compared to the infected non-treated Group II on 10th, 14th, and 30th days PI. On comparing the infected CMX and SeNPs-treated groups, a statistically nonsignificant reduction was reported in SeNPs-treated mice on the 10th day PI. However, this reduction in the SeNPs-treated group became statistically significant starting from the 14th PI with a mean oocyst count of 0.40 ± 0.21 (92.36%R) till the end of the study on the 30th day PI with the lowest mean oocyst count (0.12 ± 0.12) and the highest reduction (97.96%R). Whereas CMX-treated mice recorded a rise in the mean oocyst count (1.40 ± 0.25) and decline in percentage reduction (75.55%R) on the 30th day PI, two weeks after discontinuation of treatment, signifying recurrence of cyclosporiasis (Table 1).

**Table 1:** Fecal oocyst count retrieved from mice in the infected groups at different studied durations

Infected group Oocyst count per HPF	Non treated (G II)	CMX-treated (G III)	SeNPs-treated (G IV)	F	*p*
**6th day**					
Median (Min. – Max.)	0.65 (0.3–0.8)	0.55 (0.2–1)	0.65 (0.3–0.9)	0.070	0.932
Mean ± SD.	0.62 ± 0.19	0.58 ± 0.29	0.63 ± 0.22		
**10th day**					
Median (Min. – Max.)	1.95 (1.7–2.9)	1 (0.90–1.2)	0.8 (0.6–0.9)	38.096^*^	< 0.001^*^
Mean ± SD.	2.13 ± 0.47	1.02 ± 0.12	0.78 ± 0.12		
%R		52.34	63.28		
Significance	p_1_ < 0.001^*^, p_2_ < 0.001^*^, p_3_ = 0.360				
**14th day**					
Median (Min. – Max.)	5.25 (4.5–5.8)	0.95 (0.8–1.10)	0.35 (0.20–0.70)	474.398^*^	< 0.001^*^
Mean ± SD.	5.23 ± 0.46	0.95 ± 0.10	0.40 ± 0.21		
%R		81.85	92.36		
Significance	p_1_ < 0.001^*^, p_2_ < 0.001^*^, p_3_ = 0.015^*^				
**30th day**					
Median (Min. – Max.)	5.98 (4.3–6.2)	1.4 (1–1.70)	0.10 (0–0.30)	270.171^*^	< 0.001^*^
Mean ± SD.	5.73 ± 0.71	1.40 ± 0.25	0.12 ± 0.12		
%R		75.55	97.96		
Significance	p_1_ < 0.001^*^, p_2_ < 0.001^*^, p_3_ < 0.001^*^				

% R: Percentage of reduction of each infected treated group relative to the infected non-treated control group.

F: F for One way ANOVA test, used in comparison between more than two groups.

Post Hoc test (Tukey) is used in pairwise comparisons.

p: p-value for comparing the studied infected groups.

p_1_: p-value for comparing infected non-treated and infected CMX-treated groups.

p_2_: p-value for comparing infected non-treated and infected SeNPs-treated groups.

p_3_: p-value for comparing infected CMX-treated and infected SeNPs-treated groups.

*: Statistically significant at *p* ≤ 0.05.

#### Ultrastructural study

**Fig. 6:**
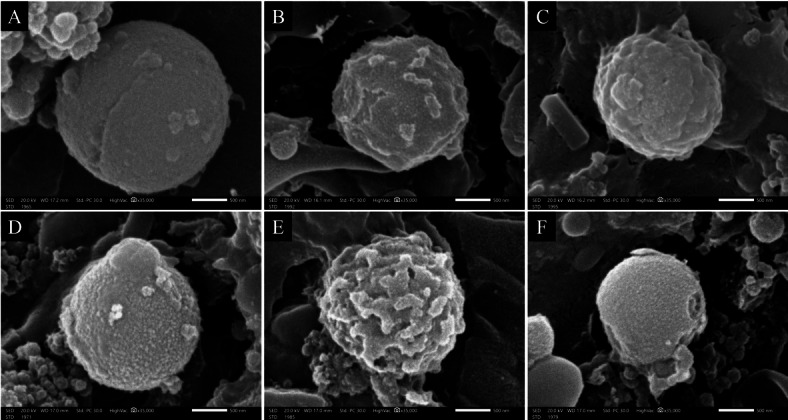
Scanning electron microscopy of *C. cayetanensis* oocysts recovered from stool of infected mice (*n* = 36): (**A**) Non-treated oocysts showing typical spherical shape with intact regular surface (x35,000); (**B** & **C**) CMX-treated oocysts revealing superficial irregularities and external protrusions, erosions and indentations (x35,000); (**D**-**F**) SeNPs-treated oocysts showed (**D**) distorted body with large protrusions (x35,000); (**E**) prominently enlarged polyps (x35,000); (**F**) shrunken body with extended surface lacerations (x35,000)

SEM examination revealed that oocysts collected from stool samples of the infected non-treated mice were generally spherical, and their surface appeared regular with fine granulations (Fig. 6A). Meanwhile, some oocysts recovered from CMX-treated mice revealed surface irregularities with superficial erosions and blebs (Fig. 6B). Other oocysts showed superficial dimples and protrusions (Fig. 6C). On the other hand, oocysts isolated from SeNPs-treated mice demonstrated severe and marked morphological and ultrastructural changes. Most of the oocysts were deformed with large surface bullae (Fig. 6D) while others revealed a completely distorted shape with numerous polyps (Fig. 6E). Some oocysts were shrunk with deep wide surface ulcerations (Fig. 6F).

#### Histopathological study

**Fig. 7:**
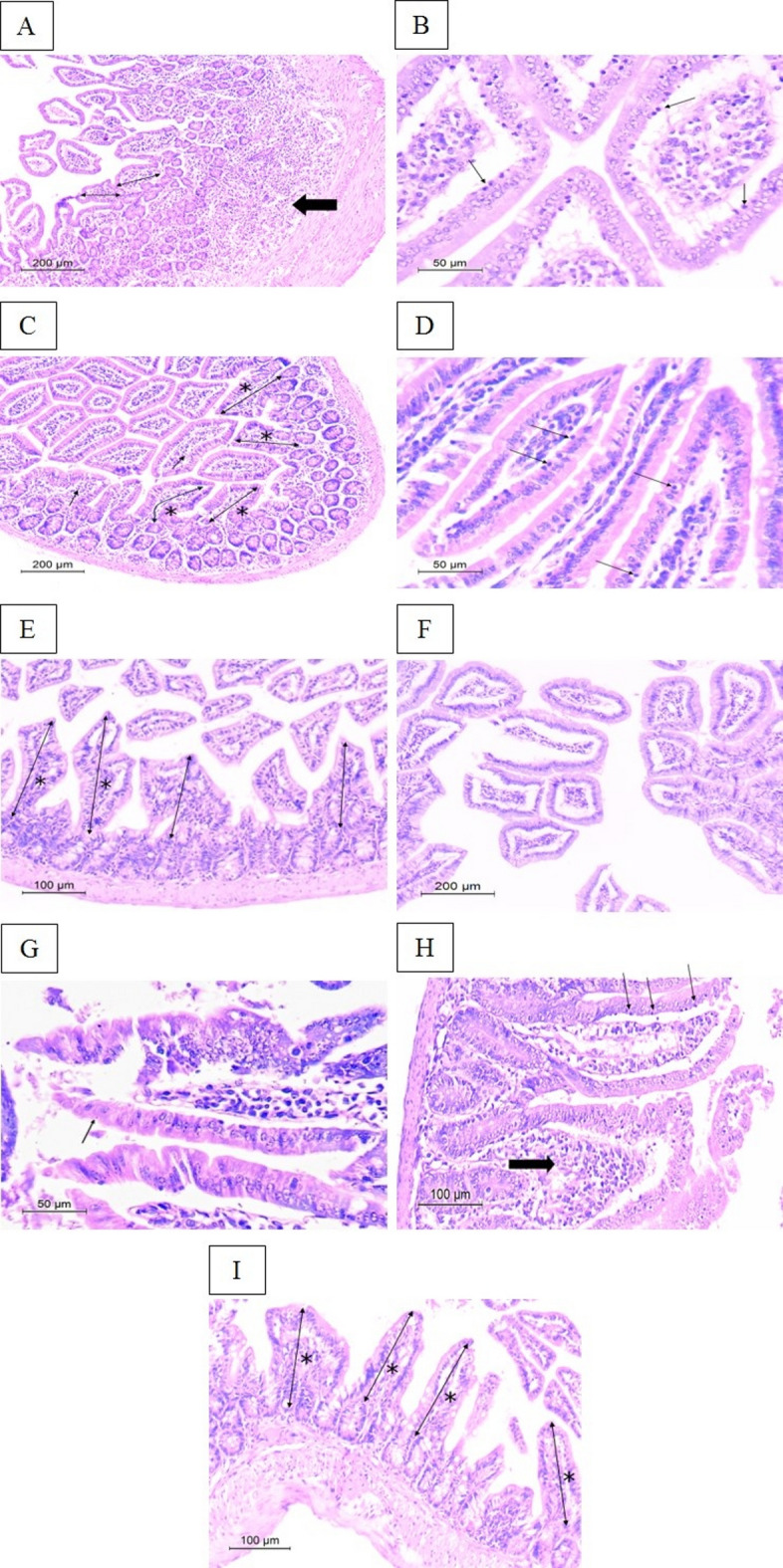
H&E-stained intestinal sections of mice in the infected groups (*n* = 36). On the 14th day PI: (A&B) Non treated mice showing (**A**) reduction in villus height (thin arrow) and dense chronic lymphoplasmacytic and neutrophilic inflammatory infiltrate in the lamina propria (thick arrow) with focal epithelial erosions (x100); (**B**) increased intraepithelial lymphocytes (thin arrows) (x400); (C&D) CMX-treated mice revealing (**C**) improved villus architecture and height (thin arrows with asterisk) with less prominent inflammatory infiltrate in the lamina propria ( short thin arrows) (x100); (**D**) detectable intraepithelial lymphocytes (thin arrows) (x400); (**E**&**F**) SeNPs-treated mice displaying (**E**) increased villus height (thin arrows) and normal lamina propria without inflammatory infiltrate (x200); (**F**) intact surface epithelium with bland epithelial nuclei and no detectable intraepithelial lymphocytes (x100). On 30th day PI: (**G**) Non treated mice still showing supranuclear *C. cayetanensis* oocyst towards the apical surface of the intestinal epithelial cell (thin arrow) (x400); (**H**) CMX-treated mice demonstrating reappearance of lamina propria oedema and inflammation (thick arrow) and marked increase in intraepithelial lymphocytes (thin arrows) (x200); (**I**) SeNPs-treated mice presenting preserved villus architecture and height (thin arrows), intact surface epithelium and lack of intraepithelial lymphocytes (x200)

On the 14th day PI, infected non-treated mice showed marked architectural disarray with evident reduction in mean villus height (136.4 ± 35.2 μm) and foci of epithelial erosions. The lamina propria revealed dense lymphoplasmacytic inflammatory infiltrate with some neutrophils (Fig. 7A and B). As regards the infected CMX-treated mice, the villous architecture was restored with a statistically significant increase in the mean villus height (202 ± 30.7 μm) with 48.09% increase compared to the infected non-treated mice. The lamina propria inflammation and focal intraepithelial lymphocytic infiltrate were decreased but still evident (Fig. 7C and D). While SeNPs-treated mice displayed intact surface epithelium showing no signs of erosions and a statistically significant increase in mean villus height (288.9 ± 17 μm) with 111.80% increase (Table 2). Interestingly, no inflammatory infiltrate was detected either in the lamina propria or epithelial cells (Fig. 7E and F).

On the 30th day PI, the infected non-treated group demonstrated similar histological changes to the 14th day PI with persistent infection (Fig. 7G). Nevertheless, the mean villus height decreased to 127.7 ± 33 μm. Conspicuously, deterioration of morphological changes was documented in the infected CMX-treated group in the form of diffuse focal surface erosions, marked inflammation in the lamina propria, and reappearance of intraepithelial lymphocytes (Fig. 7H). Furthermore, a statistically significant increase in the mean villus height (173.5 ± 14.3 μm) with 35.87% increase was recorded. There was a statistically significant decline in mean villus height on the 30th day PI, in comparison to the 14th day PI. Contrarily, SeNPs-treated group achieved profound improvement in histopathological findings, including preserved villous architecture, intact surface epithelium, and no intraepithelial lymphocytes (Fig. 7I). It is worth mentioning that the highest statistically significant increase in mean villus height (303.8 ± 13.9 μm) and the greatest %increase (137.90%) was attained in the SeNPs-treated group on the 30th day PI (Table 2).

**Table 2:** Measurement of villus height in the ileal sections of different studied infected groups on the 14th and 30th day PI

Infected group Villus height (µm)	Non treated (G II)	CMX-treated (G III)	SeNPs-treated (G IV)	F	*p*
**14th day**					
Median (Min. –Max.)	138 (93.1–176.3)	199.5 (170.8–238.2)	293.3 (264.9–304.1)	28.478^*^	< 0.001^*^
Mean ± SD.	136.4 ± 35.2	202 ± 30.7	288.9 ± 17		
% increase		48.09	111.80		
Significance	p_1_ = 0.025^*^, p_2_ < 0.001^*^, p_3_ = 0.005^*^				
**30th day**					
Median (Min. – Max.)	125.3 (90.3–169.7)	171.7 (158.4–192.3)	301 (290–323)	67.251^*^	< 0.001^*^
Mean ± SD.	127.7 ± 33	173.5 ± 14.3	303.8 ± 13.9		
% increase		35.87	137.90		
Significance	p_1_ = 0.041^*^, p_2_ < 0.001^*^, p_3_ < 0.001^*^				
t (p_4_)		3.296^*^ (0.046^*^)	3.424^*^ (0.042^*^)		

% increase: Percentage of increase of each infected treated group relative to the infected non-treated control group.

F: for One way ANOVA test used in comparison between more than two groups.

Post Hoc test (Tukey) used in pairwise comparisons.

t: for Paired t-test used in comparison between two different durations.

p: p-value for comparing the studied groups.

p_1_: p-value for comparing infected non-treated and infected CMX-treated groups.

p_2_: p-value for comparing infected non-treated and infected SeNPs-treated groups.

p_3_: p-value for comparing infected CMX-treated and infected SeNPs-treated groups.

p_4_: p value for Paired t-test for comparing between either infected CMX-treated or infected SeNPs-treated groups on 14th and 30th day PI.

*: Statistically significant at *p* ≤ 0.05.

#### Biochemical study

On the 14th day PI, all infected groups (II, III, and IV) showed a statistically significant increase in their serum MDA levels when compared to the non-infected non-treated control. On the other hand, both infected treated Groups (III and IV) verified a statistically significant decrease in the serum MDA levels in response to treatment compared to the infected non-treated Group (II). Comparing the two infected treated groups, the highest statistically significant reduction (63.46%R) in MDA level was noticed in the infected SeNPs-treated group (IV) (Table 3).

As to serum GSH levels, treatment of the infected mice with CMX and SeNPs triggered a statistically significant increase in the mean levels of GSH when compared to the infected non-treated mice with 71.43% and 300% increase, respectively. Noteworthy, the level of GSH in the infected CMX-treated mice exhibited a statistically significant decrease as compared to the non-infected non-treated mice. Meanwhile, the infected SeNPs-treated mice showed a statistically significant increase compared to mice in the non-infected non-treated (Group I) or infected CMX-treated (Group III) (Table 3).

**Table 3:** Serum MDA and GSH levels of the different studied groups on the 14th day PI

Group Marker	Non infected Non treated (G I)	Infected	Infected	Infected	F	*p*
		Non treated (G II)	CMX-treated (G III)	SeNPs-treated (G IV)		
**MDA** (nmol/ml)						
Median (Min. – Max.)	6.17 (5.82–6.39)	29.26(25.14–31.3)	17.27(14.96–19.4)	10.38(9.84–11.25)	273.859^*^	< 0.001^*^
Mean ± SD.	6.13 ± 0.22	28.6 ± 2.3	17.22 ± 1.65	10.45 ± 0.58		
%R			39.79	63.46		
p_0_		< 0.001^*^	< 0.001^*^	< 0.001^*^		
Significance		p_1_ < 0.001^*^, p_2_ < 0.001^*^, p_3_ < 0.001^*^				
**GSH **(mg/dl)						
Median (Min. – Max.)	2.88 (2.15–3.28)	1.2 (1–1.32)	1.79 (1.47–3.26)	4.88 (3.87–5.23)	70.282^*^	< 0.001^*^
Mean ± SD.	2.83 ± 0.37	1.19 ± 0.11	2.04 ± 0.66	4.76 ± 0.46		
% increase			71.43	300		
p_0_		< 0.001^*^	0.028^*^	< 0.001^*^		
Significance		p_1_ = 0.017^*^, p_2_ < 0.001^*^, p_3_ < 0.001^*^				

% R: Percentage of reduction of each infected treated group relative to the infected non-treated control group.

% increase: Percentage of increase of each infected treated group relative to the infected non-treated control group.

F: F for One way ANOVA test used in comparison between more than two groups.

Post Hoc test (Tukey) is used in pairwise comparisons.

p: p-value for comparing the studied groups.

p_0_: p-value for comparing non-infected non-treated and each infected group.

p_1_: p-value for comparing infected non-treated and infected CMX-treated groups.

p_2_: p-value for comparing infected non-treated and infected SeNPs-treated groups.

p_3_: p-value for comparing infected CMX-treated and infected SeNPs-treated groups.

*: Statistically significant at *p* ≤ 0.05.

#### Safety study

**Fig. 8:**
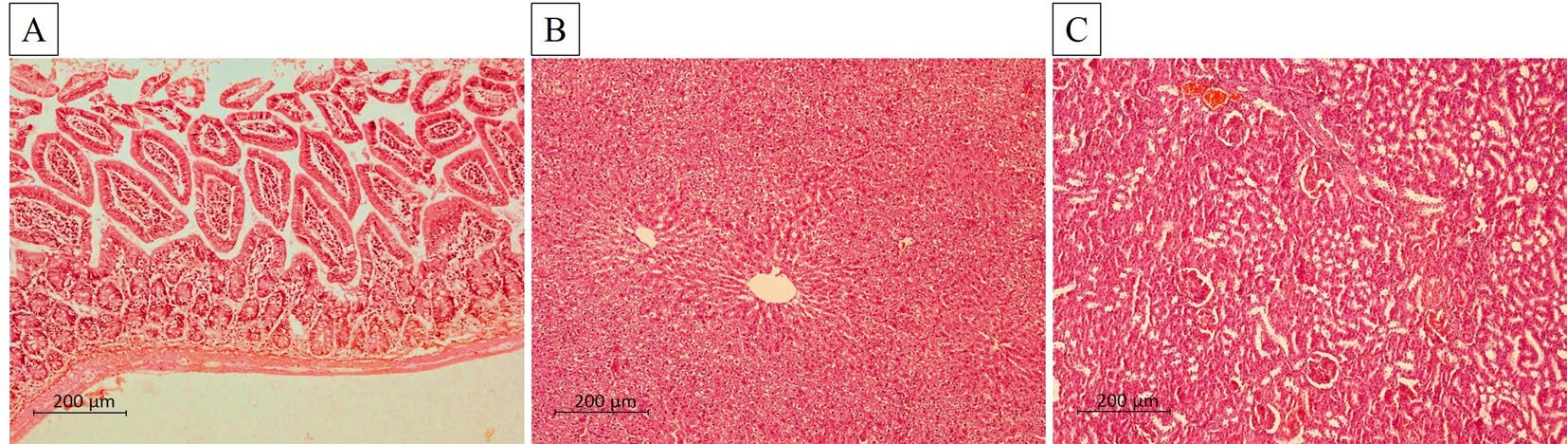
H&E-stained ileum, liver and kidney sections of mice in non-infected SeNPs-treated mice (*n* = 6): (**A**) Ileal section revealing preserved villus architecture, intact surface epithelium and normal lamina propria with no signs of oedema or inflammation (x100); (**B**) liver section displaying normal hepatic architecture with hepatocytes arranged in cords radiating from central veins (x100); (**C**) Kidney section exhibiting well-preserved renal corpuscles, distinct organised renal tubules with no inflammation or necrosis (x100)

Daily clinical observation revealed no deaths or changes in the general behavior of mice in the two studied groups. As regards body weight, a statistically non-significant difference was recorded in SeNPs-treated mice compared to their non-infected non-treated control (Table 4). Concerning the biochemical analysis, there were non-statistically significant differences in serum levels of ALT, AST, and creatinine in animals treated with SeNPs as compared to non-infected non-treated one (Table 5). Furthermore, the H&E-stained ileum, liver, and kidney sections of mice treated with SeNPs exhibited normal preserved architecture without any detrimental effects in comparison to non-infected non-treated one (Fig. 8).

**Table 4:** The effect of SeNPs on the body weight in grams of non-infected treated mice compared to their non-treated control

Non infected group Body weight (grams)	Non treated	SeNPs-treated	t	*p*
**At the beginning**				
Median (Min. – Max.)	22.95 (21.50–24)	22.60 (21–23.60)	0.715	0.491
Mean ± SD.	22.92 ± 0.97	22.50 ± 1.04		
**After 7 days**				
Median (Min. – Max.)	25.20 (23.30–26.70)	24.60 (22.80–25.50)	1.027	0.328
Mean ± SD.	25.07 ± 1.28	24.40 ± 0.95		
p_0_	< 0.001^*^	< 0.001^*^		

t: for Student t-test used to compare the two studied groups.

p: p-value for comparing the two studied groups.

p_0_: p value for Paired t-test for comparing between either non-infected non treated or non-infected SeNPs-treated groups at the two studied durations.

*: Statistically significant at *p* ≤ 0.05.

**Table 5:** Serum levels of ALT, AST, and creatinine in non-infected SeNPs-treated mice compared to their non-infected non-treated control

Non infected group Marker	Non treated	SeNPs-treated	t	*p*
**ALT** (IU/L)				
Median (Min. – Max.)	26.47 (25.34–28.55)	28.05 (24.80–29.13)	0.902	0.388
Mean ± SD.	26.69 ± 1.14	27.45 ± 1.71		
**AST** (IU/L)				
Median (Min. – Max.)	22.76 (20.11–25.52)	21.98 (20.64–24.18)	0.720	0.488
Mean ± SD.	22.87 ± 1.85	22.18 ± 1.41		
**Creatinine** (mg/dl)				
Median (Min. – Max.)	0.69 (0.55–0.80)	0.77 (0.59–0.83)	1.026	0.329
Mean ± SD.	0.69 ± 0.09	0.74 ± 0.09		

t: for Student t-test used to compare the two studied groups.

p: p-value for comparing the two studied groups.

*: Statistically significant at *p* ≤ 0.05.

## Discussion

For the treatment of human cyclosporiasis, concerted efforts have been exerted to address recorded shortcomings of CMX, including serious adverse effects, drug resistance and high recurrence rate^6^. Based on their high potency against other relevant coccidian parasites, eco-friendly SeNPs were utilised as an efficient, safe nanotherapeutic against cyclosporiasis, especially in immunosuppressed cases.

In the current study, some outlined challenges, such as human host specificity, high inoculum requirement, and limited chronicity, were faced in establishing *C. cayetanensis* infection in a murine model^3^. To overcome the model constraints and build on prior studies using the same animal model, a high inoculum dose was used. In addition, weekly immunosuppression was induced to maintain infection for 30 days and study its recurrence^35–37^.

It is worth mentioning that the association of bacterial biomolecules with SeNPs, proved via EDX analysis, empowered them with considerable stabilisation and self-functionalization by serving as capping/dispersing agents, without an additional functionalization step that is commonly applied by physicochemical synthesis methods^50,51^. Visualised TEM micrographs depicted uniform small-sized spherical nanoparticles with a high surface area-to-volume ratio, which provide additional active areas for cooperation with parasitic molecules, facilitating their penetration^52,53^. Regarding the functional traits of SeNPs, the coupling of different functional groups conjugated at their surface ascertained the participation of bacterially derived biomolecules in both precursor reduction and capping process, ensuring their colloidal stability. Further transformation of colloidal SeNPs into the amorphous aggregated Se forms is prevented, which eventually facilitates their anti-parasitic activity efficiently^21,30^. Wherein, the advantageous zeta-potential value (−22.2 mV) revealed the high electrostatic repulsion among SeNPs and Brownian motion, hence, minimizing the chance for both agglomeration and settling rates, ultimately long-term stability^37,45^. Moreover, the negative charge of SeNPs could be ascribed to the negatively charged bacterial entities, which were tightly conjugated with SeNPs, furnishing them with functionality and stability by acting as a dispersing agent^21^. While the PDI value of 0.45 implied the monodispersity and homogeneous distribution of SeNPs^45^.

Parasitologically, a lower percentage of reduction in oocyst shedding in CMX-treated mice (Group III) than SeNPs-treated ones (Group IV) at different evaluation times was recorded. However, the re-increase in mean oocyst count two weeks following cessation of CMX treatment ensured an infection recurrence^37^. The higher antiparasitic activity of biogenic SeNPs could be attributed to their ability to induce parasitic programmed cell death by disrupting transmembrane electron transport, impeding adenosine triphosphate synthesis, and hampering DNA replication^31,54,55^. Sabella et al.. (2014) postulated rapid internalisation of metal NPs into infected cells via active processes in a lysosome-enhanced Trojan horse effect^56^. Arafa et al.. 2023 speculated that the association of SeNPs in cytoplasmic vacuoles might indicate their nuclear penetration and interaction with DNA^30^.

The bilayered *C. cayetanensis* oocyst wall is considered a strong, robust defence against adversarial host and environmental threats^57^. The abnormal surface changes in oocysts retrieved from infected CMX-treated mice could be ascribed to its biological activity as a folate inhibitor interfering with DNA and protein biosynthesis, which consequently affected the oocyst wall. Kindred apoptotic hallmarks were reported by a preceding study^37^. Meanwhile, the structural alterations in oocyst wall integrity retrieved from SeNPs-treated mice could be explained by various mechanisms encompassing electrostatic interactions and overproduction of reactive oxygen species (ROS) that eventually led to a decrease in oocyst burden. The robust electrostatic interaction between bio-SeNPs and the surface of *C. cayetanensis* oocyst could bring on protein degradation of its wall^54,58^. Besides serving as antioxidants in normal cells, SeNPs could dramatically cause catastrophic ROS-mediated oxidative stress in infected cells, disrupting them from their surface to core and finally inducing apoptosis^10,54,58^. Ifigen et al. reported that bio-SeNPs induced surface changes and leakage of cellular contents of *Toxoplasma gondii*^58^.

At the histopathological level, *C. cayetanensis* infection triggered substantial oxidative-induced damage and inflammation in the intestine of immunosuppressed mice extending to the 30th day PI. These findings were asserted by other studies that linked persistent infection to its chronic nature in the immunosuppressive state^35,37^. Despite apparent improvement in intestinal architecture and inflammation witnessed in infected CMX-treated mice, recurrence of infection was still detected on the 30th day PI. A Similar pattern was observed by another study that ascribed the recurrence of infection after ceasing CMX treatment to its incomplete resolution^37^. Conversely, impressive amelioration in histopathological intestinal findings was observed in SeNPs-treated mice. Even after discontinuation of treatment, restoration of normal intestinal architecture and restraining inflammation without recurrence were protracted to the 30th day PI. This was consistent with Alkhudhayri et al., who reported that SeNPs had abated intestinal damage and inflammation induced by the intracellular coccidian, *Eimeria papillate*, in a murine model^59^.

During intracellular coccidian infection, a status of redox imbalance dominates due to the upsurge of MDA levels and subsequent depletion of endogenous GSH, leading to tissue pathology and damage^30,33,60,61^. The statistically significant lowering of serum MDA level following SeNPs treatment was attributed to the impact of Se as a constituent of selenoenzymes, glutathione peroxidases, increasing their antioxidant defence activity, which in succession led to upregulation of other antioxidant enzymes that utilised the active sites of SeNPs to detoxify the generated ROS^10,30,33^. Intriguingly, biogenic SeNPs possessed great antioxidant activity and high safety that potentially protect healthy cells from induced oxidative stress^10^. Other scholars reported restoration of serum GSH level in rats treated with SeNPs and mitigation of induced oxidative damage^62^.

A head-to-head comparison between SeNPs and CMX demonstrated the superiority of SeNPs in providing better anti-*Cyclospora* activity, extending to the 30th day PI without recurrence of infection in immunosuppressed mice. Due to their high antioxidant potential, SeNPs have been shown to ameliorate intestinal inflammation and damage. Thus, greenly synthesized SeNPs hold a promise for combating drug-resistant opportunistic parasites.

## Conclusions

Based on the current study, the greenly synthesized SeNPs were hailed as promising anti-*Cyclospora* nanotherapeutics as they induced evident antiparasitic activity, ameliorated intestinal pathology, and prevented recurrence of the infection. This is the first preclinical study on the potential therapeutic efficacy of biosynthesized SeNPs against experimental cyclosporiasis in immunocompromised murine models. Further studies are needed to test the efficacy of other doses of SeNPs and verify their fundamental pharmacokinetic and dynamic properties.

## Data Availability

All generated or analysed relevant data are included in the manuscript and are available with the corresponding author upon reasonable request.
